# Craniofacial and Dental Anomalies of a Patient Carrying Two MicroRNA Variants: A Proof‐Of‐Concept Case Report

**DOI:** 10.1002/ccr3.70137

**Published:** 2025-04-07

**Authors:** Camilla Grenga, Rosanna Guarnieri, Martina Mezio, Adriana Assunta De Stefano, Gabriella Galluccio, Roberto Di Giorgio, Agnese Giovannetti, Antonio Pizzuti, Viviana Caputo, Ersilia Barbato

**Affiliations:** ^1^ Department of Oral and Maxillofacial Sciences, School of Dentistry “Sapienza” University of Rome Rome Italy; ^2^ Clinical Genomics Laboratory Fondazione IRCCS Casa Sollievo Della Sofferenza, S. Giovanni Rotondo (FG) San Giovanni Rotondo Italy; ^3^ Department of Experimental Medicine “Sapienza” University of Rome, Clinical Genomics Laboratory, Fondazione IRCCS Casa Sollievo Della Sofferenza, S. Giovanni Rotondo (FG) San Giovanni Rotondo Italy

**Keywords:** craniofacial anomalies, dental anomalies, microRNA, MIR146A, MIR182, polymorphisms

## Abstract

Craniofacial and dentofacial anomalies often result from disruptions in embryogenetic processes driven by genetic alterations. Dental development involves complex interactions between coding and non‐coding genes, orchestrated by a network of signaling pathways. Next Generation Sequencing (NGS) has identified genes, particularly in the WNT signaling pathway, associated with dental anomalies. MicroRNAs (miRNAs), small non‐coding RNA molecules, play a crucial role in post‐transcriptional regulation. Variants in miRNAs, such as in MIR146A, have been linked to various craniofacial pathologies. A 10‐year‐old female with a class II molar malocclusion and maxillary constriction was examined. Clinical and radiographic assessments revealed impacted cuspids (both maxillary canines and the right mandibular canine), odontoma, and root resorption. Genetic analysis showed that the patient carried two variants in MIR146A (rs2910164) and MIR182 (rs76481776). The patient exhibited skeletal anomalies including class II ponticulus posticus and sella turcica bridging. The proof‐of‐concept study incorporates relevant literature discussing the molecular basis of dental anomalies, suggesting to take into account the potential functional role of miRNAs. Previous research has associated MIR146A polymorphisms with various diseases, highlighting the need for a comprehensive understanding of genetic influences on craniofacial development. This case report presents craniofacial and dental anomalies in a patient carrying two miRNA variants. Understanding the genetic basis of dental anomalies, particularly the role of miRNAs, holds promise for future advancements in orthodontics, enabling personalized diagnostics and prognostics.

**Summary**

- Variants in microRNAs may contribute to unique craniofacial and dental anomalies, suggesting their potential role in the genetic basis of orthodontic conditions.
- Understanding these links could pave the way for advancements in precision diagnostics and personalized orthodontic treatment.

## 1 Introduction

Craniofacial and dentofacial anomalies are the result of alterations in embryogenetic processes, caused by DNA alterations and/or the expression of both coding and non‐coding genes [1].

Since the formation of dental elements is a delicate and dynamic process, the causes and manifestations of dental anomalies are very complex [2].

A finely tuned balance between signaling ligands, their receptors, inhibitors and transcription factors regulate different aspects of odontogenesis including size, number, and shape. Therefore, alterations in these pathways lead to fundamental changes in odontogenesis and the appearance of dental anomalies [3].

Thanks to Next Generation Sequencing (NGS) methods, several genes have been identified that, when mutated, may contribute to canine maxillary impaction. In particular, variants have been found in genes coding for proteins with a role in the WNT signaling pathway [4, 5, 6, 7, 8].

In recent years, extensive research has explored the connection between microRNAs and human diseases. MicroRNAs (miRNAs) are small RNA molecules that play a crucial role in regulating gene expression. They work by a mechanism called RNA silencing, which essentially means they can turn down or turn off the expression of specific genes. Instead of causing significant, abrupt changes in gene expression, miRNAs typically make subtle adjustments. Because of this precise regulatory role, any imbalance in miRNA levels can indicate or contribute to various diseases, making them valuable biomarkers for diagnosing and understanding pathological conditions [9].

Several studies have highlighted the significant roles of specific miRNAs in dental development and maturation. For example, MIR200C regulates enamel formation, MIR21 supports alveolar bone osteogenesis, and MIR34A promotes odontogenic and osteogenic differentiation. Additionally, miRNAs like MIR29 and MIR31 modulate periodontal homeostasis and dental eruption during orthodontic tooth movement, highlighting their importance in tooth growth and movement regulation [10, 11, 12, 13].

MIR146A has been extensively studied in association with periodontal health homeostasis, inflammation, periodontal ligament cell differentiation, and osteoblastic differentiation [14, 15, 16, 17].

More recently, MIR146A variants have been studied. Indeed, polymorphisms in microRNAs can alter pre‐miRNA processing or maturation and influence their target specificity, resulting in phenotypic differences [18].

MIR182, a member of the MIR183 family, has been implicated in various biological processes, including development, cell differentiation, and apoptosis [19].

Recent research has suggested that MIR182 plays a role in the regulation of osteogenic differentiation and bone formation. Furthermore, MiRNA182 has been extensively analyzed in literature, in association with tumor pathologies and ocular pathologies. The rs76481776 variant of MIR182 has been reported in literature in association with primary open‐angle glaucoma and Behçet's disease [20, 21, 22]. While the provided case report briefly mentions the presence of a polymorphism in miR182 (rs76481776) in the patient, the specific impact of this variant on dental anomalies is not extensively discussed.

The importance of understanding the biological and molecular bases of dental anomalies is strongly linked to future perspectives in the orthodontic field in order to guarantee the clinician diagnostic improvement, thanks to the detection of predictive factors, and prognostic improvement, based on the concepts of customized orthodontics and the integration of data from omics sciences with the support of artificial intelligence [23, 24, 25]. Particularly, in the field of precision medicine, genomic characterization and the study of post‐transcriptional regulation processes of coding genes occupy a very wide position.

The goal of this proof‐of‐concept case report is to detail the craniofacial and dental anomalies in a patient with two variants in MIR146A and MIR182, suggesting to take into account the potential functional role of miRNAs in the genetic basis of orthodontic conditions.

## 2 Diagnosis and Etiology

### 2.1 Clinical Examination

A 10‐years old female was referred to the department of Orthodontics at Sapienza, University of Rome, in Italy from a general practitioner colleague.

**FIGURE 1:**
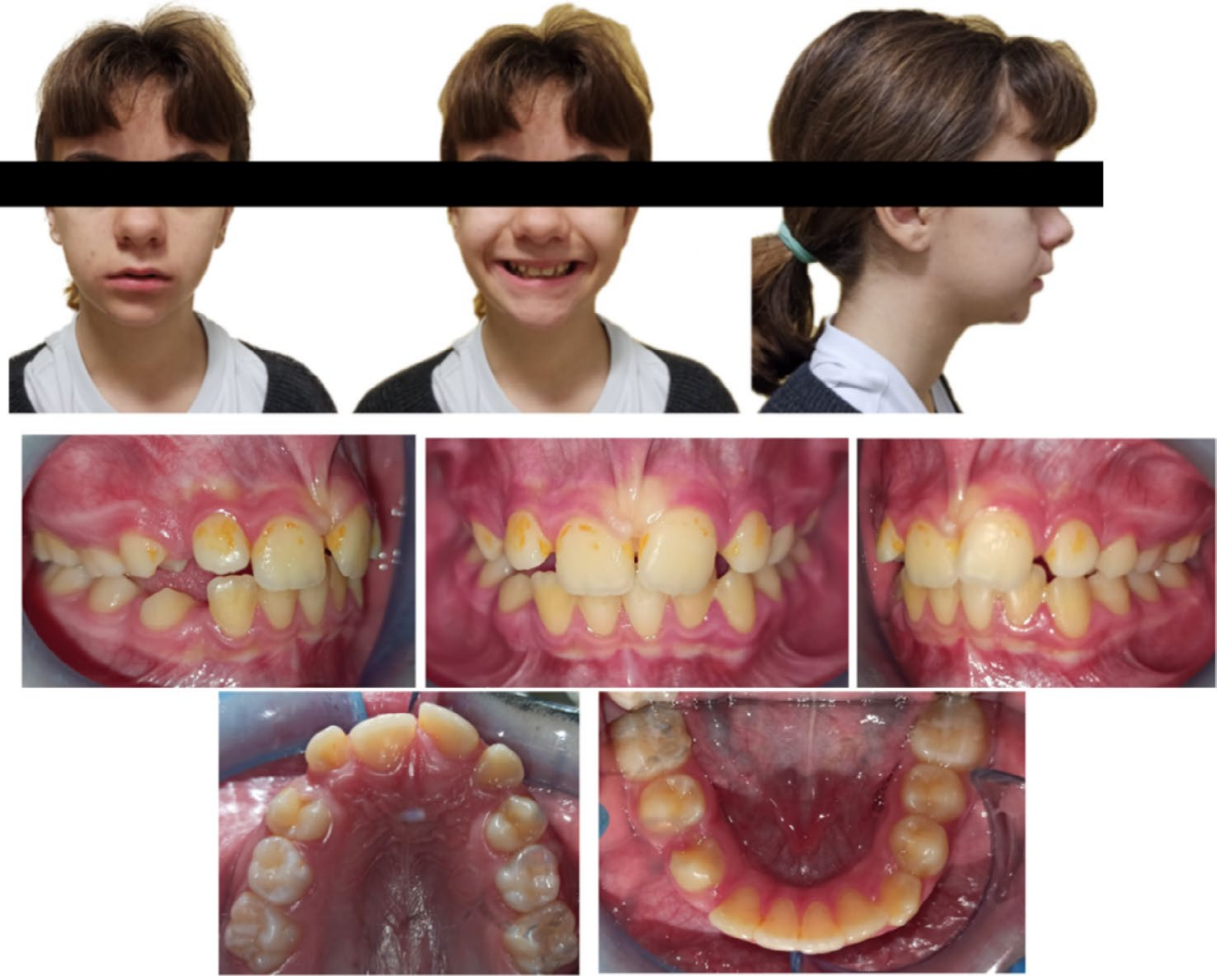
Extraoral and intraoral photographs.

At the extraoral examination the patient presented no facial asymmetries, a convex profile with a retrognathic mandible in a mesiofacial type with brachyfacial tendency. The lips were incompetent at rest (Figure 1).

Intraoral examination revealed a class II molar malocclusion with a constricted maxilla in late mixed dentition.

In the upper arch both cuspids were absent with no space for eruption. Upper midline was centered with the face midline. Labial frenum was abnormal and hypertrophied.

In the lower arch midline was non coincident with the upper one with a deviation to the right. Lower right cuspid was absent with no space for eruption.

Oral hygiene was poor. Indeed, the evaluation of the plaque index according to Silness & Löe gave a result of 2.5, indicating an abundance of plaque both in the sulcus and in the gingival margin and on the visible surface of the tooth [26] (Figure 1). The gingiva was hypertrophy.

The parents reported that no family members had experienced orthodontic problems or needed orthodontic therapy; furthermore the patient had been treated previously with a resin expansion plate superiorly and inferiorly with a lingual arch anchored to the lower first molars.

### 2.2 Radiographic Examination

**FIGURE 2:**
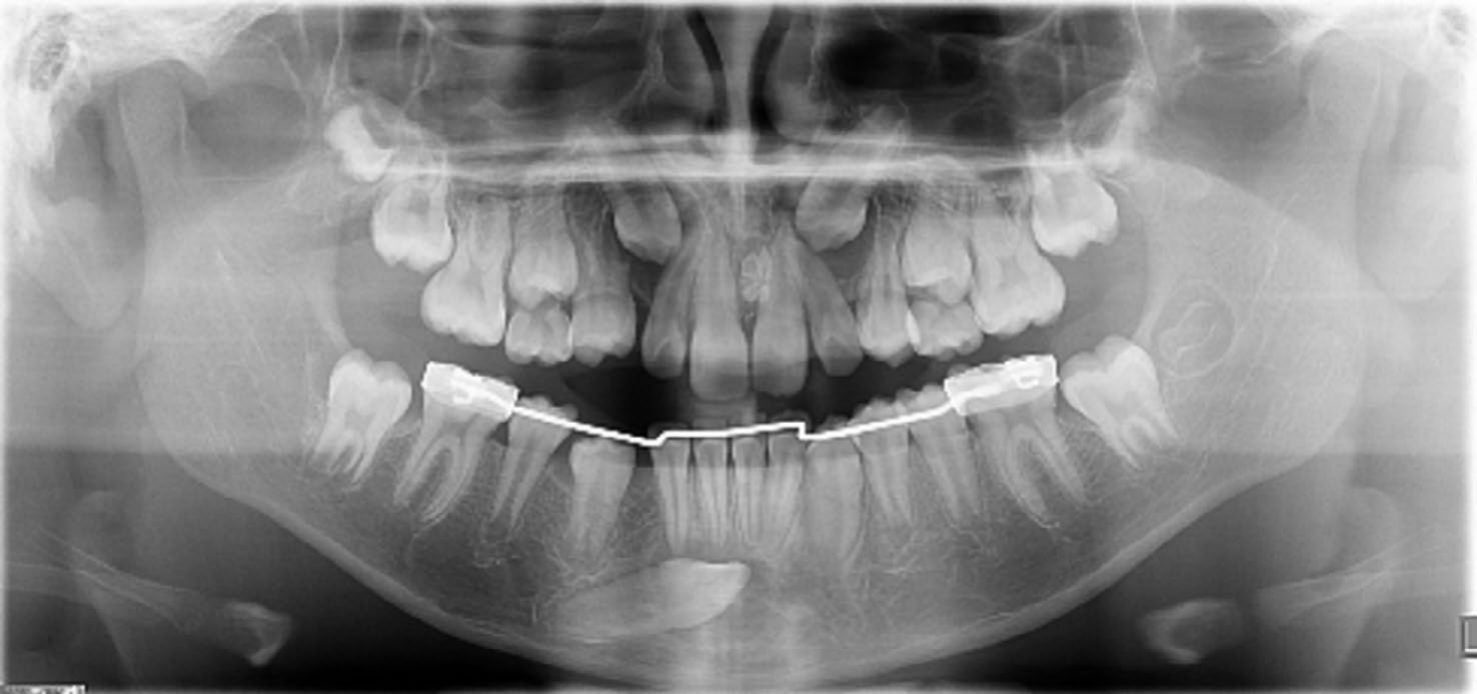
Panoramic radiography.

Orthopantomography detected the presence of all permanent teeth with both upper cuspids at a very high position in the eruptive path (Figure 2).

The position of the upper impacted canines was evaluated using the criteria proposed by Ericson and Kurol, later simplified by Baccetti et al. According to these parameters, the measured values for the right canine (13) were: angle *α* of 30° and distance from the occlusal plane of 29 mm. For the left canine (23), the values were: angle *α* of 35° and distance from the occlusal plane of 31.2 mm. Both upper canines were classified as being in sector 3 [27, 28]. Additionally, the lower left canine (33) has crossed the midline of the mandibular interincisal region, indicating significant displacement.

The close proximity between the lateral incisors and the bicuspids also contributed to the evident lack of space for eruption as seen radiographically. A large follicular space was observed around the crowns of both maxillary canines, characterized by a notable radiolucency bordered by a radiopaque rim. Root resorption is present at the apical third of the 14th, adjacent to the enlargement of the follicular sac of the upper right maxillary canine. In the second sextant, an irregular radiopaque mass was detected between the roots of the upper central incisors. In the lower arch, the right cuspid was positioned anomalously, deviating from its eruptive path, located under the roots of the incisors with the crown extending beyond the midline (transmigration of type II [29]). Enlargement of the follicular space was also observed in this case. A lingual arch, previously used in another clinic and removed before our visit, was also noted.

**FIGURE 3:**
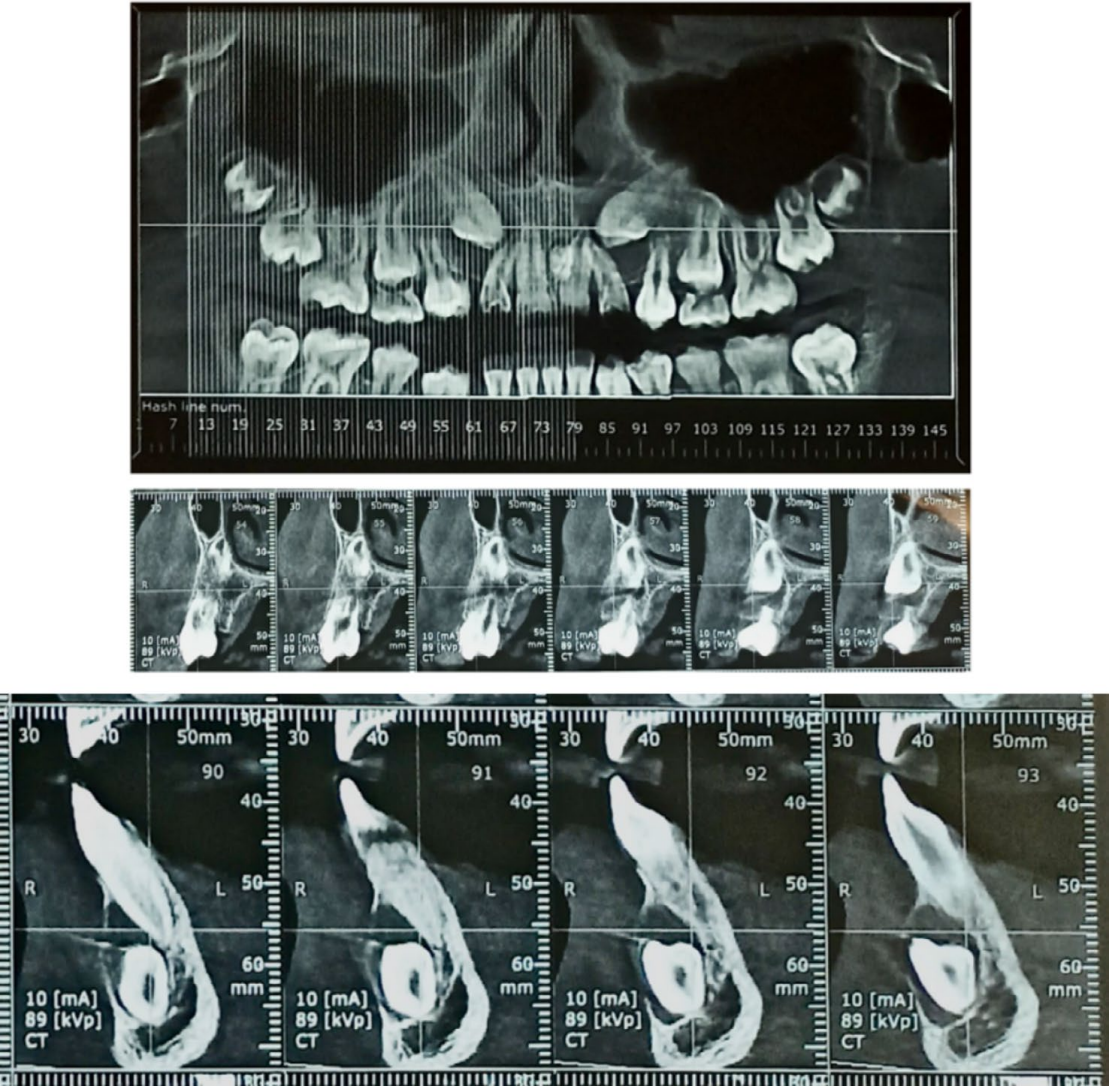
CBCT scans regarding root resorption in the first upper right bicuspid and erosion of the labial cortical concerning the cuspid.

CBCT confirmed the elevated position of the upper canines along with enlargement of the follicular space and root resorption in the first upper right bicuspid (Figure 3).

The detailed three‐dimensional evaluation confirmed the position of the radio‐opaque neoformation behind the roots of the upper central incisors and the presence of a rim delimiting the neoformation with a diagnostic suspicion of interincisal odontoma.

Regarding the lower right cuspid, CBCT also confirmed enlargement of the follicular space with erosion of the labial cortical at the crown (Figure 3).

Lateral cephalometric radiograph showed a partial calcification of sella turcica (class II of sella turcica bridging) and an incomplete or partial calcification of the atlanto‐occipital ligament (class II of ponticulus posticus) [30].

Cephalometric analysis revealed a protrusion of the upper jaw (SNA 86°) with a normal mandible (SNB 79°) in a skeletal class II malocclusion (ANB 7°, Wits Appraisal 2 mm). Vertical parameters showed a normodivergent pattern (Go‐Gn to SN 34°, FMA 28°).

**FIGURE 4:**
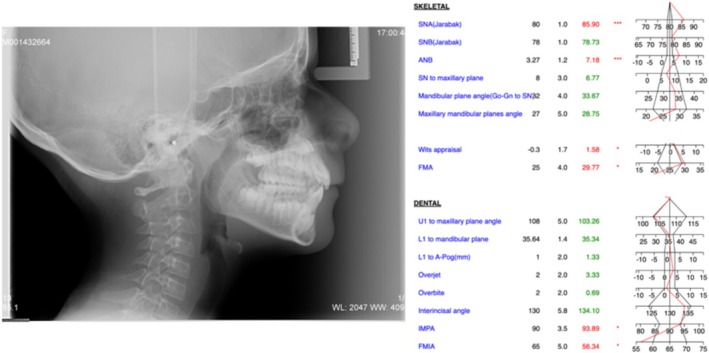
Lateral cephalometric radiograph and cephalometric analysis.

Upper and lower incisors position and inclination were normal (U1 to maxillary plane 103°, IMPA 93°) as well as overjet and overbite (3 and 1 mm, respectively) (Figure 4).

Dental and skeletal anomalies identified during clinical and radiographic examination are summarized in Table 1.

**TABLE 1:** Clinical and radiographic anomalies.

Dental characteristics	Skeletal characteristics
Impacted cuspids (13, 23, 43)	Class II ponticulus posticus
Interincisor odontoma	Class II sella turcica bridging
Root resorption (14)	

## 3 DNA Extraction and Sample Analysis

After clinical evaluation, a patient saliva sample was collected and genomic DNA was extracted by using the ORAcollect kit OC‐175 (DNA Genotek Inc. Ottawa, Canada), as per manufacturer's protocol.

Sample was included in a sequencing screening of a preliminary screening of 5 miRNAs, selected from literature as involved in tooth eruption anomalies [31]. miRNAs were PCR‐amplified by using AmpliTaq Gold DNA Polymerase (Thermo Fisher Scientific Inc., Waltham, MA, USA) and custom primers. Sanger sequencing was performed by using the ABI BigDye Terminator Sequencing Kit V.3.1 as per the manufacturer's protocol and an ABI Prism 3500 Genetic Analyzers (Applied Biosystems, Foster City, CA, USA). Sequence electropherograms were analyzed by using ChromasPro V.1.7.5 (Technelysium Pty Ltd., Brisbane, Australia).

All clinical and genetic research received approval from the regional Ethical Review Board of the “Umberto I” General Hospital of Rome (Ref. 3781) and was carried out in accordance with the ethical principles outlined in the Declaration of Helsinki. The patient who participated in this study provided written informed consent.

## 4 Results

As a proof‐of‐concept case report, we investigated the complete genomic sequence of 5 miRNAs belonging to a list of miRNAs potentially involved in odontogenesis [31]. Patient was found to be heterozygous for two SNVs (Single Nucleotide Variants): rs2910164 (C/G) located in MIR146A and rs76481776 (C/T) in MIR182. rs2910164 is located in the seed sequence of MIR146A, which is the sequence through which the miRNA target mRNAs. The variant is highly frequent in the general population (C = 30% and G = 70% according to gnomAD genomes 4.1.0 database) and has a MiRLog score of 0.27, indicating that it is unlikely to be deleterious [32]. However, the variant has been reported in literature in association with different diseases, including the risk of non‐syndromic supernumerary teeth [33]. According to miRNASNPv3 the variant is predicted to upregulate miRNA expression and to modify mature miRNA target genes (gain of 2417 and loss of 2902 targets) [34]. MIR146A has been extensively studied in literature, mostly in association to tumor [35]. Moreover, it has been recently suggested that it could regulate the expression of genes which play a role on tooth eruption (e.g., *RUNX2*) [19].

The rs2910164 variant localized in MIR146A has been studied in association with various pathologies and malformations, summarized in Table 2.

**TABLE 2:** Unraveling rs2910164 variant's role in pathologies and malformations.

*MIR146A* polymorphism *rs2910164*	
1. Ovarian cancer	Liu et al. [36]
2. Vulnerable atherosclerotic plaque	Song et al. [37]
3. Atherogenic dyslipidemia	Liu et al. [38]
4. Type 2 diabetes mellitus	Gholami et al. [39].
5. Lung cancer	Wang et al. [40]
6. Psoriasis	Gong et al. [41]
7. Cleft palate	Pan et al. [42]
8. Digestive system cancer	Lv et al. [43]
9. Melanoma	Anelli et al. [44]
10. Bladder cancer	Zhang et al. [45]

The SNV rs76481776 is located closed to the of 3′ extremity of MIR182. The variant C > T observed in the patient showed a frequence in the general population of 5.8% (C = 94.2% and T = 5.8% gnomAD genomes 4.1.0) and showed a MiRLog score of 0.04, indicating that it is predicted to be neutral. Of note, in the same genomic position, another variant had been reported in the general population, rs76481776 C > G, observed only in one individual. The variant rs76481776 (T allele), found also in our patient, has been reported in literature in association to primary open angle glaucoma [20]. According to miRNASNPv3 the variant is predicted to have a mild effect on miRNA expression and it doesn't modify target genes.

MIR182 has been reported in literature in association with cancers and ocular diseases through model organisms as mouse (MGI database) [46]. Finally, this miRNA has been proposed to play a role in osteogenic differentiation of PDLCs (periodontal ligament cells [19]).

## 5 Discussion

The presented case report highlights a unique scenario of craniofacial and dental anomalies in a patient carrying two variants in MIR146A (rs2910164) and MIR182 (rs76481776).

Maxillary canines are the second most commonly impacted teeth, following third molars (wisdom teeth), with an occurrence rate of about 1%–3%. When these canines are impacted, the most common issue that arises is root resorption (RR) of the nearby teeth, particularly the lateral incisors. Root resorption of lateral incisors happens in 8.20%–89.61% of cases, and it can also affect central incisors, though less frequently, with rates ranging from 1.19% to 35.06%. In rare instances, the premolars may also experience root resorption, with reported rates between 1.8% and 11.72% [47, 48, 49, 50, 51, 52].

In literature, the widening of the dental follicle, a condition present for the 13, 23, and 33, does not seem to contribute to the resorption of the adjacent teeth but rather to their displacing [50, 53].

Considering this, our clinical case highlights the non‐mechanistic but intrinsic aspects of the subject linked to root resorption of 14. In fact, genetic predisposition has already been studied only regarding external apical root resorption and orthodontic treatment [54, 55, 56].

As several studies have highlighted the significant roles of specific miRNAs in dental development and maturation [10, 11, 12, 13] we sequenced, as a proof‐of‐concept study, the genomic sequence of the patient for 5 miRNAs potentially involved in this biological process [31]. In this case, we found two miRNA variants, that is, rs2910164 in MIR146A and rs76481776 in MIR182. Although these two variants seem not to be deleterious, MIR146A and MIR182 have been described in the literature in relation to various pathologies, including inflammatory disorders and ocular diseases, their precise mechanism of action remains partially unknown. In particular, MIR146A is known for its influence on the inflammatory response and the regulation of genes involved in dental health, such as *RUNX2*, but its exact connection to dental root erosion or other dental anomalies has not been fully elucidated. Similarly, MIR182, which plays a role in osteogenic differentiation, may contribute to the manifestation of skeletal anomalies such as canine impaction, although this link warrants further investigation. It is important to note that, as we did not perform any other sequencing experiments on the DNA of the patient, we cannot exclude the presence of other genetic variants that may be responsible for the observed phenotype. The aim of this proof‐of‐concept study is to preliminary investigate the genetic variability of miRNAs potentially involved in odontogenesis.

The associations between ponticulus posticus, sella turcica bridging, and dental anomalies have garnered attention within the realm of craniofacial studies.

Ponticulus posticus is a vertebral anomaly that forms due to ossification of the atlanto‐occipital membrane of the posterior arch of C1 and the sella turcica bridging is a calcification of the interclinoid ligament [57].

Several studies report an association between skeletal anomalies such as ponticulus posticus and sella turcica bridging and dental anomalies such as impaction of the maxillary canine, both in syndromic and non‐syndromic patients [30, 57, 58, 59, 60]. This positive association might be explained by the involvement of neural crest cells and/or hox (homeobox) genes during the early stages of development. These genetic and cellular factors play a crucial role in the formation of various structures, including the sella turcica, teeth, and cervical vertebrae. Since these structures share developmental pathways, anomalies in these pathways can lead to related developmental issues. Key pathways involved include the Wnt Signaling Pathway; the Bone Morphogenetic Protein (BMP) Signaling; the Fibroblast Growth Factor (FGF) Signaling; the Notch Signaling Pathway, which regulates cell differentiation processes; and the Hedgehog Signaling Pathway, essential for regulating the growth and patterning of various body parts [61].

Therefore, it would be very interesting, in our opinion, to further investigate the potential functional role of miRNAs also in the bridging of the sella turcica or ponticulus posticus.

## 6 Conclusion

This proof‐of‐concept case report presents craniofacial and dental anomalies in a patient carrying two miRNA variants. Understanding the genetic basis of dental anomalies, particularly the role of miRNAs, holds promise for future advancements in orthodontics, enabling personalized diagnostics and prognostics.

## Author Contributions

**Camilla Grenga:** conceptualization, writing – original draft, writing – review and editing. **Rosanna Guarnieri:** methodology, supervision, validation, writing – review and editing. **Martina Mezio:** investigation, methodology, visualization. **Adriana Assunta De Stefano:** validation, visualization. **Gabriella Galluccio:** data curation, supervision. **Roberto Di Giorgio:** resources, visualization. **Agnese Giovannetti:** conceptualization, methodology, project administration. **Antonio Pizzuti:** conceptualization, data curation. **Viviana Caputo:** conceptualization, investigation, methodology. **Ersilia Barbato:** resources, supervision, validation, visualization, writing – review and editing.

## Ethics Statement

Ethical approval for this study was obtained from the Ethics Committee of the Sapienza University at Policlinico Umberto I, under approval number Ref. 6834. The research was conducted in accordance with the Declaration of Helsinki.

## Consent

Written informed consent was obtained from the patient for the publication of this case report and any accompanying images, in accordance with the ethical standards outlined in the manuscript.

## Conflicts of Interest

The authors declare no conflicts of interest.

## Permission to Reproduce Material From Other Sources

Permission to reproduce figures, tables, or other materials from previously published works was obtained from the copyright holders where necessary.

## Data Availability

All data generated or analyzed during this study are included in this published article and its supplementary information files. Additional data may be made available from the corresponding author upon reasonable request.
